# Blood biomarkers predict conversion from cognitively stable to mild cognitive impairment or Alzheimer's disease in Down syndrome at 16‐month follow‐up in ABC‐DS

**DOI:** 10.1002/alz.71659

**Published:** 2026-07-14

**Authors:** Fan Zhang, Melissa Petersen, Mark Mapstone, James Hall, Benjamin Handen, Brad Christian, Elizabeth Head, Herminia Diana Rosas, Florence Lai, Joseph H Lee, Sharon J Krinsky‐McHale, Frederick A. Schmitt, Jordan Harp, Christy Hom, Ira T. Lott, Sigan Hartley, Shahid Zaman, Beau M. Ances, Lauren Ptomey, Jeffrey M. Burns, Ann D. Cohen, Adam M. Brickman, Sid E. O’ Bryant, for the Alzheimer's Biomarkers Consortium‐Down Syndrome (ABC‐DS) Investigators*

**Affiliations:** ^1^ Institute for Translational Research University of North Texas Health Science Center Fort Worth Texas USA; ^2^ Department of Family Medicine University of North Texas Health Science Center Fort Worth Texas USA; ^3^ Department of Neurology University of California Irvine California USA; ^4^ Department of Psychiatry University of Pittsburgh Pittsburgh Pennsylvania USA; ^5^ Department of Medical Physics and Psychiatry University of Wisconsin Madison Madison Wisconsin USA; ^6^ Department of Pathology & Laboratory Medicine, Department of Neurology University of California Irvine California USA; ^7^ Departments of Neurology and Radiology, Massachusetts General Hospital Harvard Medical School Charlestown Massachusetts USA; ^8^ Department of Neurology, Massachusetts General Hospital Harvard Medical School Charlestown Massachusetts USA; ^9^ Taub Institute for Research on Alzheimer's Disease and the Aging Brain Columbia University Irving Medical Center New York New York USA; ^10^ Gertrude H. Sergievsky Center Columbia University Irving Medical Center New York New York USA; ^11^ Department of Epidemiology, Mailman School of Public Health Columbia University New York New York USA; ^12^ Department of Neurology, Neurological Institute Columbia University Irving Medical Center New York New York USA; ^13^ Department of Psychology New York State Institute for Basic Research in Developmental Disabilities Staten Island New York USA; ^14^ Department of Neurology University of Kentucky Lexington Kentucky USA; ^15^ Department of Psychiatry & Human Behavior University of California Irvine California USA; ^16^ Department of Pediatrics University of California Irvine California USA; ^17^ Department of Human Development and Family Studies University of Wisconsin–Madison Madison Wisconsin USA; ^18^ Department of Psychiatry University of Cambridge Cambridge Cambridgeshire UK; ^19^ Department of Neurology Washington University School of Medicine St. Louis Missouri USA; ^20^ Department of Internal Medicine University of Kansas Medical Center Kansas City Kansas USA; ^21^ Department of Neurology University of Kansas Medical Center Kansas City Kansas USA; ^22^ Department of Radiology University of Pittsburgh Pennsylvania Pittsburgh USA; ^23^ Taub Institute for Research on Alzheimer's Disease and the Aging Brain, Department of Neurology, Vagelos College of Physicians and Surgeons Columbia University New York New York USA

**Keywords:** Alzheimer's disease, batch effect correction, blood biomarkers, Down syndrome, early detection, feature selection, machine Learning, proteomics, Support Vector Machine

## Abstract

**INTRODUCTION:**

Individuals with Down syndrome (DS) face high risk for Alzheimer's disease (AD), yet presymptomatic detection of cognitive decline is hindered by lifelong intellectual disability.

**METHODS:**

Using data from the Alzheimer's Biomarker Consortium–Down Syndrome (ABC‐DS), blood samples from 246 participants were analyzed, yielding 404 longitudinal observations (45 Converters, 359 Stable) collected at 0, 16, and 32 months were analyzed. A Support Vector Machine was trained on 25 plasma biomarkers spanning neurodegeneration, inflammation, and vascular health, along with demographic factors (age, sex, ethnicity, karyotype, apolipoprotein E [*APOE* ε4]). Batch‐effect correction and feature selection were applied, resulting in 13 key markers.

**RESULTS:**

The refined model achieved 92.4% sensitivity, 59.9% specificity, and an area under the curve (AUC) of 77.9%, accurately identifying individuals at risk of cognitive decline up to 16 months before clinical progression.

**DISCUSSION:**

This multi‐domain, blood‐based machine learning approach demonstrates that plasma biomarkers are valuable non‐invasive tools for early detection and risk stratification of cognitive decline in DS.

## Highlights

- Developed a blood‐based machine learning model to predict conversion from cognitively stable to mild cognitive impairment (MCI) or Down syndrome–Associated Alzheimer's disease (DS‐AD) over 16‐month intervals.
- Analyzed 246 participants from the Alzheimer's Biomarker Consortium–Down Syndrome (ABC‐DS) cohort with repeated blood samples at 0, 16, and 32 months, yielding 404 longitudinal observations (45 Converters, 359 Stable).
- Integrated 25 multi‐domain plasma biomarkers (neurodegenerative, inflammatory, and vascular) and demographic variables (age, sex, ethnicity, karyotype, apolipoprotein E [*APOE* ε4]) into a Support Vector Machine classifier.
- Applied batch effect correction and feature selection to optimize model performance, identifying a final set of 13 key biomarkers.
- Model performance: sensitivity 92.44%, specificity 59.86%, area under the curve (AUC) 77.88%.
- Findings highlight that multi‐domain blood biomarkers can non‐invasively predict cognitive decline in DS, with potential implications for early detection and risk stratification.

## 1 BACKGROUND

Given that Alzheimer's disease (AD)–related neurodegeneration begins decades before clinical symptoms emerge, early detection is critical for timely intervention and the development of effective disease‐modifying therapies. However, current diagnostic approaches, including neuroimaging and cerebrospinal fluid (CSF) biomarkers, are often expensive, invasive, and not widely accessible. This has led to a growing interest in blood‐based biomarkers as a more practical and scalable alternative for predicting future cognitive decline associated with AD at the earliest stages of the disease.^1^, ^2^

Individuals with Down syndrome (DS) have a 90% lifetime risk for developing AD due to the triplication of chromosome 21, including triplication of the amyloid precursor protein (*APP*) gene and other chromosome 21–related factors that contribute not only to amyloid beta (Aβ) accumulation but also to downstream tau pathology and neurodegeneration.^3^, ^4^ Nearly all individuals with DS exhibit AD‐related neuropathology by the age of 40 years, and the majority develop clinical dementia by their mid‐50s. Despite the heightened risk of AD in individuals with DS, predicting clinical progression to mild cognitive impairment (MCI) or AD–related dementia remains a significant challenge due to individual variability in age at onset, variability in premorbid cognitive ability, the complexity of tracking disease progression, and the limited availability of accessible non‐invasive biomarkers. Addressing these challenges requires the development of accurate and accessible screening tools tailored to this high‐risk population.

**RESEARCH IN CONTEXT**

1. **Systematic review**: We searched PubMed and related databases for studies combining neurodegenerative, vascular, and inflammatory biomarkers to predict cognitive decline in Down syndrome. Although individual biomarker panels have been studied, no prior work has integrated neurodegenerative, inflammatory, and vascular biomarkers with demographic factors into a single predictive machine learning model for diagnostic conversion in this population.
2. **Interpretation**: This study demonstrates that a blood‐based, multi‐domain machine learning model combining biomarkers and demographic factors can accurately predict conversion from cognitively stable (CS) to mild cognitive impairment (MCI) or Down syndrome‐associated Alzheimer's disease (DS‐AD) over 16‐month intervals. The model incorporates batch effect correction and feature selection, resulting in a compact panel of 13 key biomarkers with strong predictive performance. This highlights the utility of blood biomarkers as a non‐invasive early detection tool in a population where cognitive testing is challenging.
3. **Future directions**: Future work should validate these findings in independent cohorts, explore additional domains such as neuroimaging, metabolomics, and genetics, and assess the clinical utility of these predictive models for early intervention and trial enrichment.

Recent advances in blood biomarker research demonstrate their potential to track AD pathology in individuals with DS.^1^, ^5^, ^6^, ^7^, ^8^, ^9^, ^10^, ^11^, ^12^, ^13^, ^14^ Several plasma biomarkers are associated with disease progression for Down syndrome–associated AD (DS‐AD), including Aβ42/40 ratio, phosphorylated tau (p‐tau181), and neurofilament light chain (NfL), all of which reflect key pathological hallmarks of DS‐AD.^5^, ^6^, ^15^ In addition, inflammatory markers such as interleukins (IL‐10, IL‐6, IL‐18), tumor necrosis factor‐alpha (TNF‐α), interferon‐γ (IFN‐γ), C‐reactive protein (CRP), and adhesion molecules (soluble vascular cell adhesion molecule [sVCAM‐1], soluble intercellular adhesion molecule [sICAM‐1]) are associated with neuroinflammation, which is a key contributor to DS‐AD pathogenesis.^16^, ^17^, ^18^ Although blood biomarkers have been useful for predicting AD progression in neurotypical adults, their utility in DS requires further validation. Machine learning methods may improve predictive performance and interpretability while addressing the population's unique biology.

Blood‐based biomarkers offer a non‐invasive, cost‐effective, and scalable approach to screening for DS‐AD.^15^ Compared to CSF and neuroimaging‐based diagnostics, blood biomarkers are more accessible and can be readily implemented in large‐scale screening efforts. However, the integration of multiple biomarkers into predictive models remains a challenge, particularly in longitudinal studies where batch effects introduced by repeated measures across different visits may introduce variability and obscure true biological signals.^19^

Batch effects are systematic technical variations that occur when samples are processed and measured in different batches, introducing non‐biological differences that can obscure true biological signals. These effects may arise from factors such as variations in sample processing times, assay performance, and environmental conditions across multiple study visits. If not properly addressed, batch effects can pose challenges in longitudinal studies, potentially leading to inaccurate conclusions.^20^, ^21^, ^22^, ^23^

The Alzheimer Biomarkers Consortium–Down Syndrome (ABC‐DS) is a longitudinal, multisite study involving ≈8–10 contributing clinical sites,^24^ aimed at identifying early biomarkers of AD in adults with DS. This initiative integrates blood‐based biomarkers, CSF, neuroimaging, and cognitive assessments to characterize disease progression in this high‐risk population. The ABC‐DS cohort provides longitudinal biomarker data, enabling researchers to track early biological changes associated with cognitive decline. The longitudinal nature of this cohort study also provides the ability to assess for potential batch effects arising from repeated measurements across different visits.

By leveraging data from ABC‐DS, this study evaluates whether plasma biomarkers can accurately predict which CS individuals will progress to MCI or DS‐AD over a 16‐month follow‐up period. In addition, considering the potential for batch effects due to repeated sampling, we evaluated the impact of pool adjustment for correcting these effects and the use of feature selection techniques to enhance the predictive models. These methodological advancements enhance the interpretability and clinical applicability of blood‐based screening tools for AD detection in DS. Our approach addresses key challenges such as batch effects and biomarker selection and demonstrates the potential of non‐invasive blood‐based biomarkers as a scalable tool to identify individuals with DS at risk for future cognitive decline.

## 2 METHODS

### 2.1 Study design and participants

Participants are enrolled and biospecimens are collected across all the ABC‐DS contributing clinical sites.^25^ Blood samples are subsequently stored at the National Centralized Repository for Alzheimer's Disease and Related Dementias (NCRAD), and samples used in the present study were processed at the Omics Core, Institute for Translational Research, University North Texas (UNT) Health Fort Worth. ABC‐DS is an ongoing study that evaluates adults with DS at 16‐month intervals. The methods have been previously published elsewhere.^24^, ^25^ Briefly, inclusion criteria for ABC‐DS include (1) age 25 years and older; (2) either estimated intelligence quotient (IQ) ≥30 or mental age (MA) of 36 months (for participants with MCI, premorbid IQ ≥30 or MA of 36 months); (3) karyotype of full trisomy 21, partial trisomy, or mosaic DS; (4) reliable study partner; (5) provision of legally valid consent and assent; (6) agreement of study partner and clinician that the participant is able to cooperate with the protocol tasks; and (7) adequate visual and auditory acuity to complete neuropsychological testing.^24^ Exclusion criteria include (1) diagnosis of DS‐AD; (2) any significant disease or unstable medical/psychiatric condition that could affect neuropsychological testing at Cycle 1; (3) presence of a motor or sensory impairment that could interfere with neuropsychological testing and for those undergoing neuroimaging; (4) claustrophobia; (5) contraindication for undergoing a magnetic resonance imaging (MRI) at Cycle 1 or problems with blood draws at Cycle 1; and (6) pregnancy, breast feeding at Cycle 1.^24^ All ABC‐DS participants undergo an informed consent process as part of the institutional review board–approved protocols and provided informed consent or assent to participate in the study.

As part of the ABC‐DS study protocol, participants undergo a comprehensive evaluation that includes a clinical interview with collection of demographic information, neuropsychological assessment, blood draw, and neuroimaging. Participants are classified as CS when they are without cognitive or functional decline.^24^ Participants are classified with MCI when some cognitive or functional decline is determined over and above what would be expected for their age on neuropsychological assessment and through informant report but not enough to impact daily living skills.^24^ Participants are classified as having dementia (DS‐AD) when they have substantial declines in both cognitive functioning and daily living skills as determined through neuropsychological assessment and informant report.^24^

This current analysis aims to understand the transition from cognitively stable aging to dementia in individuals with DS (DS‐AD) and to identify factors predicting progression. Participants were assessed at multiple time points, with blood samples and cognitive assessments collected at 0 months, 16 months, and 32 months. We examined two longitudinal prediction intervals. First, biomarker and demographic data collected at the initial visit (0 months) were used to predict diagnostic status at the 16‐month follow‐up. Second, data collected at the 16‐month visit were used to predict diagnostic status at the 32‐month follow‐up. For each interval, participants were classified by consensus diagnosis relative to the starting point of that interval. Only individuals who were CS at the beginning of each interval were included in the conversion analysis. In the 0‐ to 16‐month interval, participants were considered Stable if they were CS at both baseline (0 months) and 16 months, and Converters if they were CS at baseline but progressed to MCI or DS‐AD at 16 months. In the 16‐ to 32‐month interval, participants were considered Stable if they remained CS at both 16 and 32 months, and Converters if they were CS at 16 months but progressed to MCI or DS‐AD at 32 months. Individuals with MCI or DS‐AD at the start of a given interval were not included in the corresponding conversion analysis. The study included 246 participants with blood biomarker assessments at 0, 16, and 32 months. A total of 404 interval observations were included in the analysis, comprising 240 observations from the 0‐ to 16‐month interval and 164 observations from the 16‐ to 32‐month interval. Among these observations, 45 were classified as Converters and 359 as Stable. Demographic data, including age, sex, ethnicity, karyotype, and apolipoprotein E (*APOE*) ε4 status, were collected for all participants.

Blood samples were collected at each study visit and processed according to standardized protocols.^2^, ^26^ Plasma biomarkers were analyzed using two different platforms, the electrochemiluminescence (ECL) using Meso Scale Discovery (MSD; https://www.mesoscale.com) and the single molecule array (SIMOA) using Quanterix. Both MSD and SIMOA assays were run utilizing commercially available kits and run per vendor and kit recommendation and protocol. All material (reagents, antibodies, etc.) utilized to capture the assays were obtained from the respective commercially available kits. A total of 25 biomarkers (shown in Table 1) were assessed, including neurodegenerative AD‐specific markers, including amyloid‐β (Aβ), total tau (t‐tau), phosphorylated tau at threonine 181 (p‐tau181), and neurofilament light chain (NfL); inflammatory markers such as C‐reactive protein (CRP) and tumor necrosis factor‐α (TNF‐α); vascular markers such as soluble intercellular adhesion molecule‐1 (sICAM‐1); and additional markers including α‐2‐macroglobulin (A2M) and fatty acid‐binding protein 3 (FABP3). All biomarker measurements were performed in duplicate, with quality control measures applied, including outlier removal and log transformation for normalization.

**TABLE 1:** Demographic, genetic, and plasma biomarker characteristics of the cohort.

Characteristic	Overall, *N* = 404^a^	Converter, *N* = 45^a^	Stable, *N* = 359^a^	*p*‐value^b^
Karyotype				>0.99
Trisomy 21	367 / 404 (91%)	42 / 45 (93%)	325 / 359 (91%)	
Mosaicism	13 / 404 (3.2%)	1 / 45 (2.2%)	12 / 359 (3.3%)	
Translocation	22 / 404 (5.4%)	2 / 45 (4.4%)	20 / 359 (5.6%)	
Other	2 / 404 (0.5%)	0 / 45 (0%)	2 / 359 (0.6%)	
*APOE* ε4				0.34
No	319 / 404 (79%)	38 / 45 (84%)	281 / 359 (78%)	
Yes	85 / 404 (21%)	7 / 45 (16%)	78 / 359 (22%)	
Age	42.53 (8.93)	50.82 (6.66)	41.49 (8.63)	<0.001
Sex				0.64
Male	211 / 404 (52%)	25 / 45 (56%)	186 / 359 (52%)	
Female	193 / 404 (48%)	20 / 45 (44%)	173 / 359 (48%)	
Ethnicity–Hispanic				0.49
No	382 / 404 (95%)	44 / 45 (98%)	338 / 359 (94%)	
Yes	22 / 404 (5.4%)	1 / 45 (2.2%)	21 / 359 (5.8%)	
A2M	0.11 (0.47)	0.33 (0.53)	0.08 (0.45)	0.001
B2M	−0.18 (0.50)	−0.06 (0.56)	−0.19 (0.50)	0.11
CRP	−1.11 (1.89)	−1.42 (1.86)	−1.07 (1.89)	0.19
Eotaxin‐3	0.61 (1.25)	0.60 (0.97)	0.61 (1.28)	0.57
FABP3	−0.25 (0.46)	−0.11 (0.39)	−0.26 (0.46)	0.017
FVII	−0.13 (0.40)	−0.15 (0.32)	−0.12 (0.41)	0.68
I‐309	−0.30 (1.14)	−0.50 (1.23)	−0.27 (1.13)	0.33
IL‐10	−0.27 (0.88)	−0.17 (0.89)	−0.28 (0.88)	0.63
IL‐18	−0.05 (0.62)	0.01 (0.52)	−0.06 (0.63)	0.43
IL‐5	−0.83 (1.56)	−0.75 (1.54)	−0.84 (1.56)	0.30
IL‐6	−0.48 (1.07)	−0.27 (1.03)	−0.50 (1.07)	0.14
IL‐7	−0.93 (1.06)	−0.81 (1.00)	−0.95 (1.06)	0.50
Aβ40	0.16 (0.39)	0.15 (0.51)	0.16 (0.38)	0.65
Aβ42	0.37 (0.37)	0.33 (0.57)	0.38 (0.34)	0.32
Total tau	−0.93 (0.74)	−0.79 (1.16)	−0.95 (0.67)	<0.001
NfL	−1.11 (0.86)	−0.42 (0.60)	−1.20 (0.85)	<0.001
p‐tau181	−1.89 (0.90)	−1.32 (1.23)	−1.96 (0.83)	<0.001
PPY	−2.26 (2.06)	−2.26 (2.23)	−2.26 (2.04)	0.85
SAA	−1.69 (1.91)	−1.58 (2.06)	−1.71 (1.89)	0.91
sICAM‐1	−0.25 (0.41)	−0.31 (0.41)	−0.24 (0.41)	0.34
sVCAM‐1	−0.24 (0.41)	−0.07 (0.43)	−0.26 (0.40)	0.004
TARC	−0.72 (1.38)	−0.42 (1.25)	−0.76 (1.40)	0.072
TNC	−0.11 (0.48)	−0.15 (0.46)	−0.10 (0.48)	0.60
TNF‐α	−0.26 (0.47)	−0.25 (0.40)	−0.27 (0.48)	0.87
TPO	−2.45 (1.39)	−2.18 (1.51)	−2.48 (1.37)	0.19

Observations were classified as converters if they progressed from CS to MCI or DS‐AD during a 16‐month interval, and as Stable if they remained cognitively stable across the interval. Values are presented as *n*/*N* (%) or mean (SD). Biomarker values represent batch‐adjusted measurements. The *p*‐values were calculated using Pearson's chi‐square tests for categorical variables and Wilcoxon rank‐sum tests for continuous variables.

^a^ *n*/*N* (%); mean (SD).

^b^ Pearson's chi‐squared test; Wilcoxon rank‐sum test.

### 2.2 Batch effect correction using pool adjustment method

Because biomarker data were collected across multiple study visits and used to predict conversion at the subsequent follow‐up, batch effects arising from differences in sample processing, assay variability, and environmental conditions could confound the results and were therefore an important consideration.^20^ To address the impact of batch effects, we applied pooled control samples to ensure consistency across biomarker measurements.^27^

Each assay plate included five pooled plasma controls to ensure consistency across biomarker measurements and to reflect the age distribution of study participants with DS. These pooled samples enabled systematic normalization across batches, enhancing the reliability of the biomarker data. The same pooled controls were used consistently across multiple visits to maintain uniformity in longitudinal analyses. All determinations were conducted in duplicate, with strict quality control measures ensuring that coefficients of variation (CVs) remained below 10% for all assays, with over 60% achieving CVs of ≤6%.

The pool adjustment method corrects batch effects by normalizing individual sample values based on the average of a designated pooled sample within each batch. Each original value is normalized by dividing it by its respective pool mean. For a raw measurement of protein *i* in batch *j* for sample *k*, $X_{i,j,k}$, the adjusted value can be calculated as follows


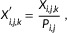

where $P_{i,j}$ is the mean value of pooled control samples for protein *i* within batch *j*, representing the batch‐specific average expression level.

### 2.3 Machine learning model development

A Support Vector Machine (SVM) classifier was developed to predict Cognitive Impairment (CI) conversion based on blood biomarkers and demographic variables. The modeling process combined the following key steps.

#### 2.3.1 Data preprocessing

Biomarker values were first adjusted using pool adjustment to correct for batch effects, followed by log transformation to reduce skewness. Outliers were then identified using the interquartile range (IQR) method, where $Q_{1}$ and $Q_{3}$ represent the 25th and 75th percentiles of each feature, respectively, and the IQR is defined as $Q_{3}-Q_{1}$. Observations falling outside the range [*Q*_1_ – 1.5 × *IQR*, *Q*_3_+1.5 × *IQR*] were considered outliers and treated as missing values prior to analysis. Missing values were imputed using a machine learning–based multiple imputation method (MLMI).^28^ Finally, all features were standardized to z‐scores to ensure comparability across scales. The dataset was evaluated using 10‐times repeated 5‐fold cross‐validation,^29^ where the data were split into five folds. Each fold was used for validation, whereas the remaining folds were used for training, and this process was repeated 10 times. This method ensures that every data point is used for both training and validation, providing a more reliable estimate of model performance by reducing variance.

#### 2.3.2 Feature selection and feature interpretability

A recursive feature elimination process was implemented to reduce dimensionality and enhance model interpretability by identifying the most relevant biomarkers.^30^ Features contributing the least to classification performance were removed iteratively using a two‐step nested cross‐validation approach.^30^ In the inner loop, a 10‐times repeated 5‐fold cross‐validation assessed model performance, whereas the outer loop employed leave‐one‐out cross‐validation to guide feature elimination. Feature importance was evaluated using an algorithm based on leave‐one‐out cross‐validation,^30^ measuring each variable's contribution to model performance. Features were iteratively removed one at a time, ensuring that only the most informative biomarkers were retained. A positive feature importance value indicated that removing the feature would degrade model performance, whereas a negative value suggested that its removal would improve predictive accuracy.

#### 2.3.3 Model training and optimization

A radial basis function (RBF) kernel was trained with the SVM classifier. Hyperparameters (C and gamma) were optimized using grid search with 5‐fold cross‐validation to maximize performance. Performance metrics, including sensitivity, specificity, and area under the receiver operating characteristic (ROC) curve (AUC), were computed to assess classification effectiveness. Nested cross‐validation was performed to mitigate potential overfitting. Performance was compared between models with and without batch adjustment and feature selection to evaluate their impact on classification performance.

### 2.4 Statistical analysis

Descriptive statistics were used to summarize participant characteristics and biomarker distributions using R (version 4.3.3) with packages from the Comprehensive R Archive Network (CRAN), including gtsummary (version 2.5.0). Group differences between Converters and Stable individuals were assessed using: (1) Wilcoxon rank‐sum tests for continuous biomarker levels, (2) chi‐square tests for categorical variables (e.g., *APOE* ε4 status, karyotype, ethnicity), and (3) Benjamini–Hochberg correction to control for multiple comparisons (false discovery rate [FDR] < 0.05).

## 3 RESULTS

### 3.1 Cohort for prediction of CI conversion

Data from 25 biomarkers collected at 0 to 16 months and 16 to 32 months in the ABC‐DS cohort^31^ were used to predict clinical disease conversion 16 months in advance. Table 1 summarizes the characteristics of these biomarkers along with five demographic covariates. The study cohort consisted of 404 observations from 246 participants, including 45 Converter and 359 Stable observations. Demographic factors, such as karyotype, *APOE* ε4 status, sex, and ethnicity, did not differ between groups (*p* > 0.05), except for age, where Converters were older than Stable individuals (50.82 vs 41.49 years, *p* < 0.001). The distribution of karyotypes among Converter observations (42/45, 93%) reflects the proportion of Converters with Trisomy 21, whereas a similar pattern was observed among Stable observations (325/359, 91%) in Table 1. This distribution is consistent with the expected karyotype distribution in DS, in which a≈95% of cases are Trisomy 21.^32^ Conversion rates were similar across karyotype groups overall (Trisomy 21: 11.4%; Mosaicism: 7.7%; Translocation: 9.1%), with no significant association between karyotype and diagnostic status (Converter vs Stable) (χ^2^ = 5.3, *df* = 6, *p* = 0.51; Table 2). Consistent findings were observed across intervals: in the 0‐ to 16‐month interval, conversion rates were 10.1% (22/217) for Trisomy 21, 12.5% (1/8) for Mosaicism, 14.3% (2/14) for Translocation, and 0% (0/1) for Other (χ^2^ = 4.24, *df* = 6, *p* = 0.64; Table 2); in the 16‐ to 32‐month interval, conversion occurred only among Trisomy 21 participants (13.3%, 20/150), with no Converters in other groups (χ^2^ = 3.78, *df* = 6, *p* = 0.71; Table 2). Several of the 25 blood biomarker levels differed between Converter and Stable individuals: A2M (*p* = 0.001), FABP3 (*p* = 0.017), tau (p < 0.001), NfL (p < 0.001), p‐tau181 (p < 0.001), and sVCAM‐1 (*p* = 0.004). Other biomarkers, including B2M, CRP, Eotaxin‐3, IL‐6, Ab40, and TNF‐α, did not differ between groups (*p* > 0.05).

**TABLE 2:** Conversion rates by karyotype.

Karyotype	Converter (0–16 mo, 16–32 mo)	Stable (0–16 mo, 16–32 mo)	Conversion Rate (0–16 mo, 16–32 mo)
Trisomy 21	42 (22,20)	325 (195,130)	11.44% (10.14%,13.33%)
Mosaicism	1 (1,0)	12 (7,5)	7.69% (12.50%,0.00%)
Translocation	2 (2,0)	20 (12,8)	9.09% (14.29%,0.00%)
Other	0 (0,0)	2 (1,1)	0.00% (0.00%,0.00%)

Conversion rates are defined as the proportion of observations within each karyotype group that progressed to MCI or DS‐AD, calculated as converter / (Converter + Stable). The denominator includes only observations that were cognitively stable at the start of the interval; those with MCI or DS‐AD at the beginning of the interval were excluded.

Abbreviation: mo, months.

### 3.2 Pool adjustment for batch effect correction

**FIGURE 1:**
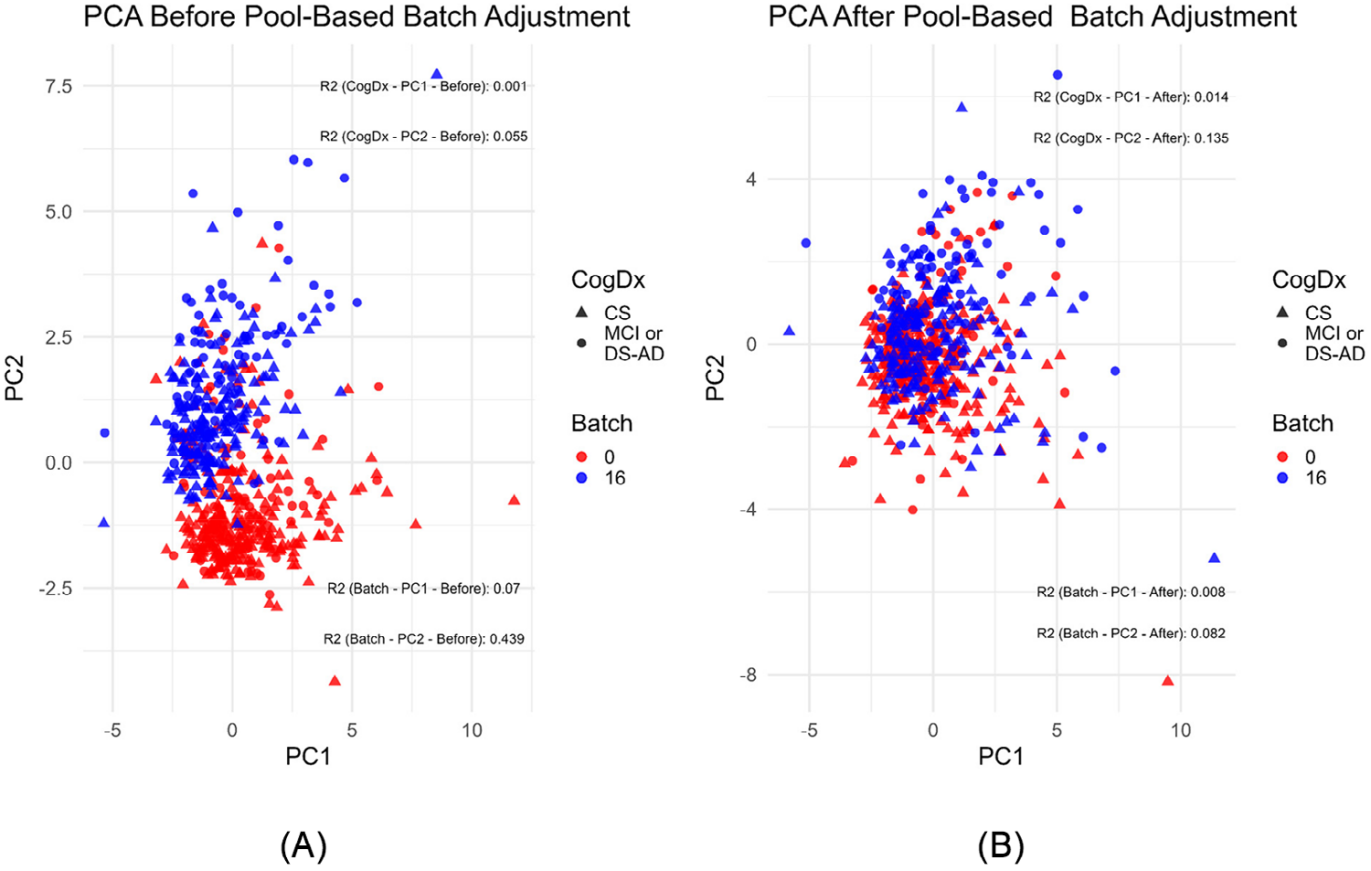
Principal component analysis (PCA) before and after pool‐based batch adjustment. PCA plots illustrate the impact of pool‐based batch adjustment on plasma biomarker data across multiple assay runs and collection timepoints. (A) Before adjustment, clustering of samples by assay batch/timepoint is observed, indicating substantial batch effects, particularly along PC2. (B) After pool‐based adjustment using pooled plasma controls across assay runs, batch‐driven clustering was markedly reduced, demonstrating improved alignment and comparability of samples across batches. The adjustment accounts for potential inter‐batch variability arising from differences in assay runs, reagent lots, instrument calibration drift, and operator‐ or time‐related effects, while preserving underlying biological variation associated with cognitive diagnosis. R^2^ values indicate the proportion of variance in PC1 and PC2 explained by batch and cognitive diagnosis before and after adjustment.

First, we used principal component analysis (PCA) to visually assess the presence of batch effects. The analysis of PCA before adjustment indicated the presence of batch effects, particularly in PC2, where batch explained a substantial portion of the variance (R^2^ = 0.439) (Figure 1a), indicating that batch differences strongly influenced the PC2. Visually, the scatter plot of the PC2 highlights a separation of the samples due to different batches, further confirming the impact of batch effects. Although the batch effect on PC1 was smaller (R^2^ = 0.07), its presence still suggests systematic differences across visits. In contrast, the influence of cognitive diagnosis (MCI and DS‐AD categorized together as cognitively impaired vs Stable) was minimal, with R^2^ values of 0.001 for PC1 and 0.055 for PC2, indicating that cognitive differences were not strongly reflected in the initial principal components.

The presence of batch effects introduced variability in biomarker measurements across multiple visits undermined the accuracy of DS‐AD prediction using machine learning. To address this issue, we applied the pool adjustment method, which effectively reduced batch effects and improved the performance of the machine learning model.

We evaluated the effectiveness of the pool‐based batch adjustment method by applying and comparing PCA before and after the correction (Figure 1b). The batch effect on PC1 was reduced with batch effect adjustment (R^2^ = 0.008), and its influence on PC2 also decreased (R^2 ^= 0.082). This finding demonstrates the effectiveness of the pool‐based adjustment in mitigating batch‐related variability. On the other hand, the cognitive diagnosis effect became slightly more evident after adjustment, with the R^2^ for PC1 increasing from 0.001 to 0.014 and the R^2^ for PC2 increasing from 0.055 to 0.135, suggesting that the adjustment preserved and possibly enhanced the ability to differentiate between cognitive groups.

### 3.3 Recursive feature elimination

To enhance model performance and identify the most relevant features, we implemented a recursive feature elimination strategy within an SVM‐based framework.^30^ The selection process began with 25 blood biomarkers and five demographic variables (karyotype, *APOE* ε4 status, age, sex, and ethnicity). We used the feature importance algorithm^30^ to evaluate each variable's contribution to model performance. The optimal feature set was determined through the two‐step nested cross‐validation approach.^30^ This rigorous selection process resulted in a final model comprising 13 features: 2 demographic covariates (age and ethnicity) and 11 blood biomarkers (A2M, CRP, FABP3, I‐309, IL‐10, sVCAM‐1, TARC, TNC, Aβ42, NfL, and p‐tau181). The final 13 markers could be categorized into three groups based on their primary roles and relevance to DS‐AD: (1) demographic/clinical Variables including age and ethnicity, (2) AD pathology/neurodegeneration markers including p‐tau181, Aβ42, NfL, FABP3, and A2M, and (3) inflammatory/vascular‐related markers including sVCAM‐1, I‐309, TARC, TNC, CRP, and IL‐10.

To further validate the importance of these features, we systematically removed each variable and reassessed model performance. In every case, classification accuracy declined compared to the optimized 13‐feature model, reinforcing the effectiveness of the feature selection process in identifying key biomarkers for early DS‐AD detection.

### 3.4 Improved model performance

The model's predictive performance improved through successive refinements, including pool adjustment and feature elimination (Table 3). Before pool adjustment, the model achieved a sensitivity of 87.51%, a specificity of 56.93%, and an AUC of 77.10% for detecting converter status. After pool adjustment, sensitivity increased to 88.62%, specificity rose to 59.66%, and AUC reached 77.28%. Further enhancement was observed with the integration of feature elimination, yielding the best performance: sensitivity of 92.44%, specificity of 59.86%, and an AUC of 77.88%. This progression highlights the effectiveness of pool adjustment and feature elimination in refining the model's predictive capabilities, particularly in reducing false negatives and improving the identification of individuals at risk of cognitive decline.

**TABLE 3:** Average Performance for testing set of 9 Converters and 71 Stables by 10 times repeated 5‐fold cross‐validation.

	Before Pool Adjustment		After Pool Adjustment		Pool Adjustment + Feature Elimination	
Predicted	CONVERTER	STABLE	CONVERTER	STABLE	CONVERTER	STABLE
CONVERTER	7.876	30.58	7.976	28.640	8.320	28.50
STABLE	1.124	40.42	1.024	42.360	0.680	42.50
Sensitivity	87.51%		88.62%		92.44%	
Specificity	56.93%		59.66%		59.86%	
AUC	77.10%		77.28%		77.88%	

Performance metrics were evaluated on the testing set consisting of 9 Converters and 71 Stables. Results represent average confusion matrix counts and model performance metrics across repeated cross‐validation runs for the before pool adjustment, after pool adjustment, and pool adjustment + feature elimination models. Sensitivity, specificity, and area under the receiver operating characteristic curve (AUC) are reported.

**FIGURE 2:**
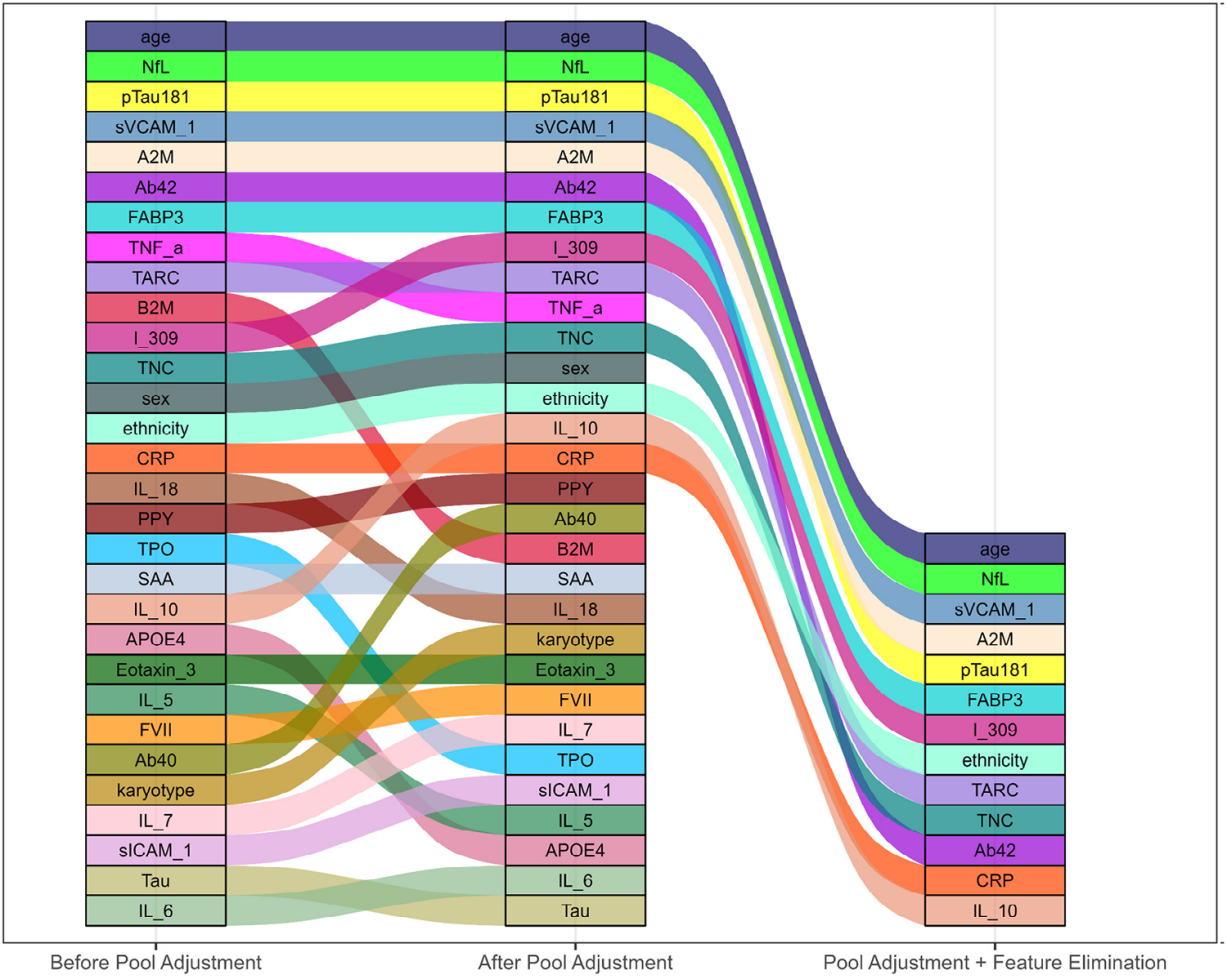
Ranking change in feature importance across Before Pool Adjustment, After Pool Adjustment, and Pool Adjustment + Feature Elimination. Sankey diagram illustrating changes in feature importance rankings across the three machine learning model stages. Pool‐based batch adjustment resulted in moderate shifts in feature rankings, reflecting reduced technical variability and refined biomarker contributions. Although the top‐ranked features (age, neurofilament light chain [NfL], phosphorylated tau 181 [pTau181], sVCAM‐1, and A2M) remained stable across models, several inflammatory and immune‐related biomarkers showed rank changes after adjustment. Recursive feature elimination further refined the model by reducing the feature set from 30 to 13 variables, retaining the most informative predictors while removing less contributory features. Persistent retention of age, NfL, sVCAM‐1, A2M, p‐tau181, FABP3, I‐309, TARC, TNC, Aβ42, CRP, and IL‐10 supports their robust contribution to predicting cognitive impairment conversion in DS‐AD. CS, cognitively stable; DS‐AD, Down syndromeassociated Alzheimer's disease; MCI, mild cognitive impairment.

To better understand the impact of pool‐based batch adjustment and recursive feature elimination on model performance, we constructed an interpretable Sankey diagram (ggsankey package v0.0.9) in Figure 2. This visualization illustrates how each feature contributed to the prediction of three models: Before Pool Adjustment, After Pool Adjustment, and Pool Adjustment + Feature Elimination. It also allows for a direct comparison of feature contributions across these stages.

Following pool adjustment, feature importance rankings experienced moderate shifts, reflecting batch effects on biomarker stability. Although the top five features (age, NfL, p‐tau181, sVCAM‐1, and A2M) remained unchanged, subtle reordering occurred among other biomarkers. Notably, IL‐10 rose in ranking from #20 to #14, suggesting an increased predictive contribution post‐adjustment. Ethnicity as a variable moved slightly upward (#14 to #13), whereas CRP dropped from #15 to #16. Some inflammatory markers, such as B2M and TNF‐α, switched positions, with B2M decreasing from #10 to #18, whereas TNF‐α moved up from #8 to #10. Minor re‐rankings also occurred among immune‐related markers such as IL‐5, IL‐6, total tau, and sICAM‐1, indicating that batch adjustment influenced their relative contributions. Although pool adjustment did not drastically alter the highest‐ranked features, it refined feature importance by mitigating batch effects, leading to a more stable and reliable ranking of predictive biomarkers.

Feature elimination further refined the model by retaining only the most informative variables, reducing the number of features from 30 in After Pool Adjustment to 13 in Pool Adjustment + Feature Elimination. Several biomarkers—including TNF‐α, B2M, IL‐18, PPY, Aβ40, SAA, karyotype, Eotaxin‐3, FVII, IL‐7, TPO, sICAM‐1, IL‐5, *APOE* ε4, IL‐6, and total tau—were removed, indicating their lower predictive contributions. However, key biomarkers such as age, NfL, sVCAM‐1, A2M, p‐tau181, FABP3, I‐309, ethnicity, TARC, TNC, Aβ42, CRP, and IL‐10 remained, highlighting their importance for classification accuracy. The elimination of non‐informative features likely enhanced model efficiency, reducing noise while maintaining strong predictive power.

Among the selected biomarkers, age, NfL, sVCAM‐1, A2M, and p‐tau181 consistently ranked among the top features across all three models, underscoring their strong predictive value for CI conversion. FABP3, I‐309, TARC, and TNC exhibited minor rank shifts but remained in the final feature set, reinforcing their relevance. Although Aβ42, CRP, and IL‐10 experienced slight downward ranking adjustments, their retention in the optimized model suggests their continued importance. Overall, the persistence of these biomarkers throughout pool adjustment and feature selection confirms their robust contribution to classification accuracy and their potential as key indicators for DS‐AD progression. In addition to these biomarker findings, an interesting observation in this cohort was that sex did not significantly contribute to model performance, whereas age was more predictive of diagnostic conversion than sex, with no significant age‐by‐sex interaction observed (ΔDeviance = 0.86, *df* = 1, *p* = 0.35; 95% confidence interval: −0.13 to 0.05) in either the pre‐pool adjustment or post‐pool adjustment analyses.

## 4 DISCUSSION

### 4.1 Batch effects and pool‐based adjustment

Our findings highlight the importance of addressing batch effects in longitudinal biomarker studies such as ABC‐DS. Although all blood‐based biomarker assays were performed at a centralized laboratory, samples were processed across multiple assay runs and submission timepoints (e.g., 0, 16, and 32 months), introducing potential non‐biological variability related to reagent lots, instrument calibration drift, assay runs, and operator‐ or time‐related effects. PCA results demonstrated that batch effects accounted for a substantial proportion of variance prior to adjustment, particularly along PC2, indicating that technical variation could substantially confound biologically meaningful disease‐related signals.

To address this issue, we implemented a pool‐based adjustment strategy that normalized biomarker measurements relative to pooled controls within each batch. Following pool‐based batch adjustment, the contribution of batch to the principal components was markedly reduced, whereas the relative contribution of cognitive diagnosis increased, suggesting improved signal‐to‐noise separation. Model performance in the testing set also improved after pool adjustment, supporting the role of batch harmonization in reducing variability and enhancing prediction accuracy. The approach provided a simple yet effective normalization strategy for preserving biological variability while minimizing technical effects when pooled controls were available.

These findings are consistent with prior studies in blood‐based biomarker research for AD, which have shown that harmonization strategies can improve reproducibility and downstream predictive performance by reducing technical variability across assay runs.^33^, ^34^ However, several limitations of the pool‐based adjustment approach should be noted. First, its effectiveness depends on the representativeness and stability of the pooled control samples across batches, and residual batch effects may persist if control materials do not fully capture run‐to‐run variability. Second, although such methods reduce systematic technical variation, they may also inadvertently attenuate subtle biological signals if batch and biological factors are partially confounded. Third, the approach assumes relatively consistent assay behavior over time and may be less effective under substantial protocol changes or major instrument upgrades.

Despite these limitations, our results support the utility of pool‐based batch adjustment in multicenter and longitudinal AD biomarker studies. In large consortia such as ABC‐DS, where data are generated across multiple timepoints and potentially heterogeneous processing conditions, robust batch harmonization is essential to ensure comparability of biomarker measurements. By reducing non‐biological variability while preserving disease‐related signal, such approaches strengthen the validity of downstream machine learning models and improve the interpretability of biomarker–clinical associations across diverse study sites and longitudinal follow‐up periods.

### 4.2 Feature selection and feasibility of blood‐based early detection

Feature selection was essential for improving predictive accuracy and model generalizability. Initial models trained using 25 biomarkers and five demographic variables (karyotype, *APOE* ε4 status, age, sex, and ethnicity) demonstrated moderate performance. To refine the model, we applied recursive feature elimination within a two‐step nested cross‐validation framework,^30^ reducing the feature set to 13 key predictors: A2M, CRP, FABP3, I‐309, IL‐10, sVCAM‐1, TARC, TNC, Aβ42, NfL, p‐tau181, age, and ethnicity. The streamlined model improved classification performance while reducing dimensionality and emphasizing biomarkers strongly linked to established AD pathology.

Our findings further demonstrate the feasibility of using blood‐based biomarkers to detect phenoconversion from stable cognition to MCI or DS‐AD ≈16 months before clinical diagnosis. Compared with invasive and costly diagnostic approaches such as CSF analysis and positron emission tomography imaging, blood‐based screening offers a scalable alternative for high‐risk populations such as individuals with DS. The elevated AD risk in DS is strongly associated with trisomy 21, including overexpression of the *APP* gene, which contributes to early amyloid accumulation; however, additional genes on chromosome 21 and elsewhere, along with downstream molecular and cellular processes, also play important roles in AD pathogenesis. Although the model demonstrated high sensitivity (92.44%) and moderate specificity (59.86%), the high prevalence of AD in older adults with DS supports its clinical utility as a screening tool. At a 12% disease prevalence, the model achieved a positive predictive value of 23.90% and a negative predictive value of 98.31%, indicating strong utility for identifying individuals unlikely to convert while supporting early monitoring and intervention in this high‐risk population.

### 4.3 Comparison with previous studies and clinical implications

Neurodegeneration and inflammation are central components of AD pathology, and our findings reinforce their importance in DS‐AD. Among the selected biomarkers, Aβ42, p‐tau181, and NfL represent established markers of amyloid deposition, tau pathology, and neurodegeneration,^35^, ^36^ whereas inflammatory and vascular markers including sVCAM‐1, I‐309, TARC, TNC, CRP, and IL‐10 emerged as strong predictors of diagnostic conversion. These results support growing evidence that chronic neuroinflammation and immune dysregulation contribute substantially to DS‐AD progression.^37^, ^38^, ^39^

Our findings are consistent with prior studies demonstrating the predictive utility of plasma biomarkers such as Aβ42/Aβ40, p‐tau181, and NfL in both sporadic and DS‐associated AD.^36^, ^40^, ^41^ However, to our knowledge, this is among the first studies in DS to integrate neurodegenerative, inflammatory, and vascular biomarkers into a single machine learning framework for prediction of conversion from cognitively stable status to MCI or DS‐AD. Previous DS studies have largely focused on associative analyses rather than predictive modeling, although some studies have examined vascular contributions together with inflammatory and AD biomarkers.^42^ Similarly, multimodal models developed in non‐DS populations often omit one or more of these biological domains or are not designed specifically for diagnostic conversion in DS. For example, the Alzheimer's Disease Neuroimaging Initiative (ADNI) proteomics and cardiovascular disease study evaluated plasma proteomic biomarkers associated with AD and cardiovascular disease over time,^43^ whereas the Cardiovascular Risk Factors, Aging and Dementia (CAIDE) model incorporated inflammatory biomarkers into AD risk prediction in a general aging cohort.^44^

These findings have important clinical and research implications. Blood‐based biomarkers provide a less invasive, more scalable, and more cost‐effective alternative to CSF and imaging‐based diagnostics, particularly for high‐risk populations such as individuals with DS. Earlier identification of at‐risk individuals may improve clinical monitoring, facilitate earlier therapeutic intervention, and enhance clinical trial enrichment strategies. In addition, the strong contribution of inflammatory markers suggests that neuroinflammatory pathways may represent promising therapeutic targets and highlights the potential of predictive models to guide future biological and therapeutic investigations.

### 4.4 Limitations and future directions

Despite the strengths of this study, several limitations should be acknowledged. (1) Limited Sample Size for Converters: Although the study included 45 Converters, larger sample sizes are needed to improve model generalizability and stability. Future studies should validate these findings in independent cohorts with greater numbers of conversion events. (2) Potential Confounders: Although demographic variables were included in the analyses, other factors such as lifestyle characteristics, comorbidities, and medication use may also influence biomarker levels and should be incorporated in future modeling efforts. (3) Longer‐Term Follow‐Up and Interval‐Based Design Considerations: This study focused on a 16‐month prediction framework, with analyses conducted across 0‐ to 16‐month and 16‐ to 32‐month intervals. Although this interval‐based approach increased the number of analyzable observations (from 246 participants to 404 interval‐level records) and better reflects real‐world repeated follow‐up assessments, it introduces within‐subject dependence due to repeated participation (*n* = 158 overlapping individuals across intervals). Although removing duplicate participants would resolve this dependence, it would reduce the effective sample size back to *n* = 246, thereby decreasing statistical power. In contrast, a 32‐month baseline‐to‐endpoint design ensures full independence but further reduces the analyzable cohort (*n* = 212), limiting model robustness. We additionally evaluated a baseline‐to‐32‐month model among cognitively stable participants at baseline. Although this approach showed slightly improved performance (sensitivity [SN] = 92.57%, specificity [SP] = 64.57%, AUC = 79.87%), it substantially reduced sample size (33 Converters and 179 Stables; testing set: 6 Converters and 35 Stables) due to the requirement for complete 32‐month follow‐up data. Overall, these results highlight a trade‐off between longer prediction horizons, statistical independence, and sample size in longitudinal modeling. (4) Integration with Other Biomarker Modalities: The current biomarker panel demonstrated high sensitivity but only moderate specificity, which may partly reflect the inclusion of markers such as NfL that capture general neurodegeneration rather than disease‐specific pathology. An ablation analysis excluding NfL yielded a modest increase in specificity (from 59.86% to 61.06%), accompanied by slight reductions in sensitivity (from 92.44% to 90.67%) and AUC (from 77.88% to 77.06%). Although these finding suggest that NfL contributes only modestly to reduced specificity, overall model performance likely reflects the combined effects of multiple biomarkers. The absence of more AD‐specific markers, such as p‐tau217 and microtubule‐binding region (MTBR)‐tau243, may further limit disease specificity; in this study, MTBR‐tau243 was not included, and p‐tau217 was excluded due to substantial missingness. Future studies integrating more specific tau biomarkers, along with complementary modalities such as genetic risk factors (e.g., *APP* and presenilin 1 [*PSEN1]* mutations) and neuroimaging measures, may enhance specificity and provide a more comprehensive characterization of disease progression.

## CONSENT STATEMENT

This study was conducted in accordance with the Declaration of Helsinki and approved by the institutional review boards (IRBs) of the participating institutions. The Alzheimer's Biomarker Consortium–Down Syndrome (ABC‐DS) cohort study received ethical approval under IRB protocols [STUDY21040184B] at the seven participating institutions. All participants or their legal guardians provided written informed consent prior to inclusion in the study. The consent process included a detailed explanation of the study's purpose, procedures, potential risks, and benefits. The privacy and confidentiality of participant data were maintained throughout the study in accordance with applicable regulations and institutional policies.

## CONFLICT OF INTEREST STATEMENT

S.E.O. has multiple pending and issued patents on blood biomarkers for detecting and precision medicine therapeutics in neurodegenerative diseases. He is a founding scientist and owns stock options in Cx Precision Medicine, Inc. No other authors reported any potential conflicts of interest. Author disclosures are available in the Supporting Information.

## Supporting information



ICMJE Disclosure Form

## APPENDIX A

### A.1

*Alzheimer's Biomarker Consortium–Down Syndrome (ABC‐DS) Investigators: Beau M. Ances, MD PhD; Howard F. Andrews, PhD; Karen Bell, MD; Rasmus M. Birn, PhD; Adam M. Brickman, PhD; Peter Bulova, MD; Jeff Burns, MD; Amrita Cheema, PhD; Kewei Chen, PhD; Bradley T. Christian, PhD; Isabel Clare, PhD; Ann D. Cohen, PhD; Eric W. Doran, MS; Tatiana M. Foroud, PhD; Benjamin L. Handen, PhD; Jordan Harp, PhD; Sigan L. Hartley, PhD; Elizabeth Head, PhD; Denise Head, PhD; Christy Hom, PhD; Lawrence Honig, MD; Milos D. Ikonomovic, MD; Sterling C Johnson, PhD; M. Ilyas Kamboh, PhD; David Keator, PhD; Julia K. Kofler, MD; William Charles Kreisl, MD; Sharon J. Krinsky‐McHale, PhD; Florence Lai, MD; Patrick Lao, PhD; Charles Laymon, PhD; Joseph Hyungwoo Lee, PhD; Ira T. Lott, MD; Victoria Lupson, PhD; Mark Mapstone, PhD; Davneet Singh Minhas, PhD; Neelesh Nadkarni, MD; Sid O'Bryant, PhD; Deborah Pang, MPH; Melissa Petersen, PhD; Julie C. Price, PhD; Lauren Ptomey, PhD; Margaret Pulsifer, PhD; Michael S. Rafii, MD PhD; Herminia Diana Rosas, MD; Frederick Schmitt, PhD; Nicole Schupf, PhD; Wayne P. Silverman, PhD; Dana L. Tudorascu, PhD; Rameshwari Tumuluru, MD; Badri Varadarajan, PhD; Michael A. Yassa, PhD; Shahid Zaman, MD PhD; and Fan Zhang, PhD.
